# The Intensity of BCL2A1 Expression Increases According to the Stage Progression of Acute Histologic Chorioamnionitis in the Extra-Placental Membranes of Spontaneous Preterm Birth

**DOI:** 10.3390/life14121535

**Published:** 2024-11-22

**Authors:** Chan-Wook Park, Eun-Mi Lee, Seung-Han Shin, Chul Lee, Jae-Kyung Won

**Affiliations:** 1 The Department of Obstetrics and Gynecology, Seoul National University College of Medicine, Seoul 03080, Republic of Korea; vita77@snu.ac.kr; 2 The Department of Pediatrics, Seoul National University College of Medicine, Seoul 03080, Republic of Korea; revival421@snu.ac.kr; 3 The Department of Pathology, Seoul National University College of Medicine, Seoul 03080, Republic of Korea; fe98134@snu.ac.kr

**Keywords:** BCL2A1, stage progression, extra-placental membranes, acute histologic chorioamnionitis, WBC, amniotic fluid, spontaneous preterm birth

## Abstract

Our prior findings showed that BCL2A1 in neutrophils is highly expressed in the extra-placental membranes (EPMs) of both the human spontaneous preterm-birth (PTB) (i.e., PTL or preterm PROM) and nonhuman-primate PTB model. However, no data exist on whether the intensity of BCL2A1 expression quantitatively increases according to the stage progression of acute histologic chorioamnionitis (acute HCA) in EPM. The objective is to investigate whether the intensity of BCL2A1 expression quantitatively increases according to the stage progression of acute HCA in EPM among spontaneous PTB cases, as measured using QuPath. The study population included 121 singleton PTBs (gestational age [GA] at delivery < 34 weeks) due to either preterm labor or preterm PROM. With digital image analysis, we calculated the percentage of BCL2A1-positive cells in immunohistochemistry according to the stage progression of acute HCA in EPMs as the primary outcome and examined the relationship between the percentage of BCL2A1-positive cells and either the GA at delivery or the amniotic-fluid (AF) WBC count as the secondary outcome. The median percentage of BCL2A1-positive cells progressively increases with the stage progression of acute HCA in EPM (group-1 vs. group-2 vs. group-3 vs. group-4 vs. group-5; 7.62 vs. 5.15 vs. 43.57 vs. 71.07; γ = 0.552, *p* < 0.000001). The percentage of BCL2A1-positive cells in EPMs and the AFWBC count shows a positive correlation (γ = 0.492, *p* = 0.000385). Moreover, the percentage of BCL2A1-positive cells in EPMs continuously decreased with increasing GA at delivery (γ = −0.253, *p* = 0.005148). In conclusion, the intensity of BCL2A1 expression increases according to the stage progression of acute HCA in EPMs and the elevation of AFWBC among spontaneous PTB cases. This finding suggests BCL2A1 in EPMs may be a promising marker and target for acute HCA.

## 1. Introduction

Acute histologic chorioamnionitis (acute HCA) is a primary pathophysiology of spontaneous preterm birth (PTB) (i.e., preterm labor [PTL] and preterm PROM) [1,2,3]. In general, acute HCA was classified based on two criteria such as stage and grade. The terminology “stage” describes the progression of disease according to advanced areas characterized by neutrophils infiltration, while the terminology “grade” indicates the intensity of infiltrating neutrophils at an advanced anatomical area [4]. The correlations that the stage progression of acute HCA, intra-amniotic inflammation (IAI), gestational age (GA) at delivery, and neonatal complications are well known for are as follows: (1) IAI is known to become more severe with the stage progression of acute HCA, as indicated by outside-in neutrophil migration within the extra-placental membranes (EPMs) [5,6]; (2) the lower the GA, the more frequent the occurrence of IAI in both PTL and preterm PROM [7,8,9]; (3) GA at delivery is earlier according to the progression of acute HCA (i.e., placenta without inflammation, inflammation restricted to chorio-decidua, and inflammation beyond chorio-decidua) [10]; and (4) moreover, early-onset neonatal sepsis, a common complication in preterm neonates, is more frequent in cases of amnionitis (a more advanced stage of inflammation in the innermost layer of the EPM) than in cases of inflammation limited to the chorio-decidua (a less advanced stage in the outermost layer of the EPM) [11]. Unfortunately, no previous report exists on the correlation between the stage and the grade that is quantitatively measured in acute HCA—assessed by the intensity at the most advanced region (i.e., the innermost area) infiltrated by neutrophils in the EPM—and the relationship between the grade that is quantitatively measured in acute HCA and IAI. There exist only a few studies reporting that the stage (or involved region) has a greater impact on IAI than the grade (or intensity) simply classified in a binary manner in acute HCA [12,13]. However, it is necessary to objectively assess not only the involved region (i.e., stage) but also the intensity (i.e., grade) in order to accurately evaluate the role of acute HCA on IAI and neonatal complications.

Although neutrophils physiologically have a brief circulatory life span (i.e., a few hours) due to apoptosis, their survival period is remarkably prolonged in inflamed tissues [14]. Initially, the Bcl-2 family protein, B-cell lymphoma 2-related protein A1 (BCL2A1), with anti-apoptotic function, was reported to interact with pro-apoptotic BCL2 proteins in the hematopoietic tissue and to be highly expressed in hematologic malignancies [15]. However, BCL2A1 has recently been recognized for its role in supporting neutrophil pro-survival and maintaining homeostasis in the context of inflammation [16]. Notably, we previously demonstrated that BCL2A1 is specifically and abundantly expressed in the neutrophils infiltrating the EPM in both human spontaneous PTB and a nonhuman-primate (NHP) PTB model induced by intra-amniotic (IA) LPS injection [17]. Moreover, treatment with a BCL2A1-specific inhibitor reduced the frequency of non-apoptotic neutrophils, as assessed by flow cytometry, in chorio-decidua cell suspension cultures from the NHP PTB model [17]. Therefore, if BCL2A1 expression can be objectively quantified in the EPM, it could not only allow for an accurate assessment of the stage progression (or involved region) and grade (or intensity) in acute HCA but also enhances its value as a therapeutic target for this condition.

QuPath (ver. 0.4.4), an open-source software platform introduced by Bankhead et al. in 2017, was specifically designed to facilitate the analysis of digital pathology images [18]. Since its release, QuPath has gained widespread adoption in pathology research for a variety of applications, particularly for its ability to enable the objective quantification of pathological findings. Its flexibility and powerful analytical tools have made it an essential resource in the field of digital pathology.

Given the expression of BCL2A1 in neutrophils infiltrating the EPM [17], it is likely that the intensity of BCL2A1 expression, as measured using QuPath, increases with the stage progression of acute HCA in the EPM and correlates positively with the severity of IAI, as indicated by the amniotic fluid (AF) WBC count. However, there are a lack of data on whether the intensity of BCL2A1 expression increases with the stage progression of acute HCA in the EPM and correlates positively with the severity of IAI among pregnant women at risk for early spontaneous PTB due to either PTL or preterm PROM. The objective of the current study is to examine this issue.

## 2. Materials and Methods

### 2.1. Study Design and Patient Population

Study population included 121 cases. The inclusion criteria of current study are as follows: <1> singleton pregnancy; <2> GA at delivery: 20.6~33.9 wks; <3> the cause of PTB, PTL or preterm PROM; <4> birth date: February 2008~January 2013; and <5> an available formalin-fixed and paraffin-embedded (FFPE) block of placenta. Moreover, the exclusion criteria of current study are as follows: <1> multifetal pregnancy; <2> GA at delivery: less than 20.6, or more than 33.9 wks, <3> the cause of PTB: maternal fetal indication (i.e., preeclampsia and fetal growth restriction); <4> birth date: before Feb. 2008, or after Jan. 2013; and <5> an unavailable formalin-fixed and paraffin-embedded (FFPE) block of placenta. With Qu-Path, we performed the quantitative analysis of BCL2A1-positive cells by immunohistochemistry (IHC) according to the stage progression of acute HCA in EPM. In some cases included in this study, transabdominal amniocentesis was conducted for the examination of microbial culture and WBC count in AF. The relationships between the percentage of BCL2A1-positive cells in the EPM and AFWBC count were investigated in 48 patients delivered within 5 d after amniocentesis. To maintain a meaningful temporal relationship, this criterion was applied between the AFWBC count and the percentage of BCL2A1-positive cells in the EPM of placenta collected at delivery. Amniocentesis was carried out after acquiring written informed consent. The IRB of our hospital approved this research (No: 2409-123-1574).

### 2.2. Clinical Characteristics and Pregnancy Outcomes

Clinical characteristics and pregnancy outcomes were obtained from a medical record review (i.e., maternal age, parity, cause of preterm delivery, gender of newborn, delivery mode, GA at delivery, birth weight, Apgar score at 1 min and 5 min, and antenatal use of corticosteroids, antibiotics, and tocolytics).

### 2.3. Diagnosis of Acute Histologic Chorioamnionitis (Acute HCA) in Extra-Placental Membranes (EPMs)

Placental tissues obtained for pathologic evaluation included a chorio-amniotic membrane roll (EPM: chorio-decidua and amnion), umbilical cord, and chorionic plate. These samples were processed to paraffin block and stained with hematoxylin and eosin (H and E) as performed in a previous study [5]. Acute HCA in EPM was defined by acute inflammatory changes, as indicated by neutrophil infiltration upon examination of chorio-amniotic membrane roll (EPM), according to the previously published criteria [5,19]. Study population was divided into 5 groups according to the stage progression of acute HCA—based on outside-in neutrophil migration—in EPM: (1) group-1, “inflammation-free EPM” (50 cases); (2) group-2, “inflammation restricted to decidua” (13 cases); (3) group-3, “inflammation restricted to the membranous trophoblast of chorion and the decidua” (25 cases); (4) group-4, “inflammation in the connective tissue of chorion but not amnion” (20 cases); and (5) group-5, “amnionitis” (13 cases).

### 2.4. Immunohistochemistry of Extra-Placental Membranes (EPMs)

IHC for BCL2A1 was performed as previously described [17]. Briefly, paraffin-embedded EPM blocks were sectioned into 4 μm thick sections and subjected to antigen retrieval by microwave boiling in citrate buffer, followed by incubation with anti-human BCL2A1 (Cat. No. LS-b450; 1:200 dilution; LSBio, Lynwood, WA, USA) in 10% normal horse serum/0.2% Tween-20 at 4 °C overnight. For indirect detection, a peroxidase conjugated goat anti-rabbit IgG antibody (PI-1000-1; 1:200 dilution; Vector Lab. Newark, CA, USA) was incubated for 30 min at room temperature. Staining is visualized using a DAB substrate. The BCL2A1-stained slides were scanned using the SCN400F Slide scanner (Leica Microsystems, Wetzlar, Germany).

### 2.5. Digital Image Analysis

Anti-BCL2A1 staining patterns of the EPM were assessed by the open-source quantitative pathology analysis program QuPath (ver. 0.4.4) [20]. The anti-BCL2A1-stained EPM slides were uploaded into the QuPath software as a Brightfield (H-DAB) image. Initially, a new EPM image was created manually by random selection of three regions with the similar area at the most advanced inflammatory region (i.e., the innermost area) infiltrated by neutrophils in EPM; R1 (placental margin edge), R2 (middle portion), and R3 (ruptured margin edge), with a measured size of ≈40,000 μm^2^ in one section, were examined, and the average number of anti-BCL2A1-positive cells across the three regions within a section was determined. To detect anti-BCL2A1-positive cells, “Positive Cell Detection” was performed using a single-intensity threshold parameter of 0.2 based on the previously reported criteria [21].

### 2.6. Amniotic Fluid (AF)

AF was cultured for the evaluation of IA infection and analyzed for the assessment of IAI based on white blood cell (WBC) count (cells/mm^3^) according to the methods previously described [22,23].

### 2.7. Statistical Analysis

The comparison of continuous variables and proportions were performed with the Kruskal–Wallis test and Pearson’s chi-square test, respectively, for the intergroup difference (Table 1). Moreover, we used 1-way analysis of variance (ANOVA) followed by a Tukey post hoc test for the comparisons of continuous variables and Fisher’s exact test with Bonferroni’s correction for the comparisons of proportions (Table 1). After performing an ANOVA, when significant differences are found among the means of three or more independent groups, the post hoc Tukey test is used to conduct pairwise comparisons to pinpoint where these differences lie. Fisher’s exact test with Bonferroni correction is used to compare proportions in categorical data across small groups, identifying where differences lie while controlling for type I error in multiple comparisons. Spearman’s rank correlation test was used to investigate the relationship between the stage progression of acute HCA and the percentage of BCL2A1-positive cells in EPM between the AFWBC count and the percentage of BCL2A1-positive cells in EPM and between GA at delivery and the percentage of BCL2A1-positive cells in EPM. Spearman correlation analysis is generally used for measuring the strength and direction of the association between two ranked (ordinal) variables. Data were analyzed using SPSS Statistics 20.0. Statistical significance was defined as a *p* < 0.05.

## 3. Results

### 3.1. Clinical Characteristics and Pregnancy Outcomes

Group-1, group-2, group-3, group-4, and group-5 are present in 41.3% (50/121), 10.7% (13/121), 20.7% (25/121), 16.5% (20/121), and 10.7% (13/121) of cases, respectively (Table 1). Table 1 demonstrated the GA at delivery and birth weight were significantly lower (γ = −0.413 and *p* = 0.000003 and γ = −0.358 and *p* = 0.000055 in Spearman’s rank correlation test, respectively), and the Apgar score at 5 min < 7 and the antenatal use of tocolytics were significantly more frequent according to the stage progression of acute HCA in the EPM (*p* = 0.008 and *p* = 0.0074 in linear-by-linear association, respectively). As an unexpected finding, the frequency of male newborns was significantly higher in group-5 compared to group-4 (*p* < 0.05, Fisher’s exact test with Bonferroni’s correction) (Table 1).

### 3.2. Determination of the Percentage of BCL2A1-Positive Cells Using QuPath Digital Image Analysis According to the Stage Progression of Acute HCA in EPM

**Figure 1:**
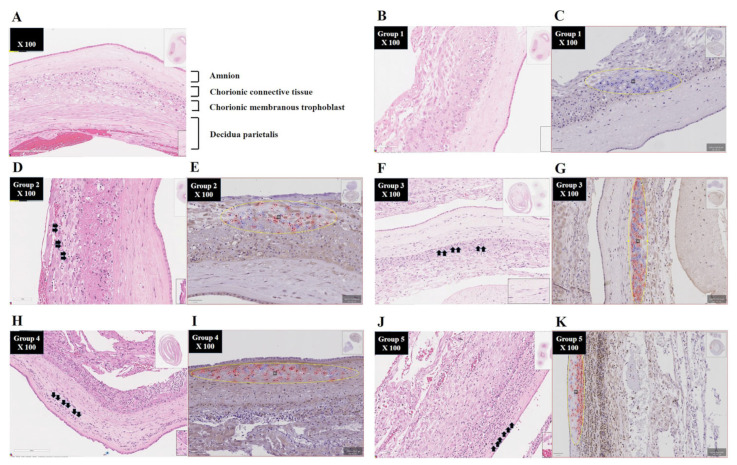
(**A**) Normal histology ((**H**,**E**) stained histologic section) of EPM. This image is based on the magnification setting × 100. Amnion, the connective tissue of chorion, the membranous trophoblast of chorion, and decidua parietalis are sequentially shown downward. (**B**–**K**) Representative (**H**,**E**) stained images (**B**,**D**,**F**,**H**,**J**) and QuPath IHC BCL2A1 images (**C**,**E**,**G**,**I**,**K**) according to the stage progression of acute HCA in EPM. The stage progression of acute HCA in EPM is as follows: (**B**,**C**) group-1: “inflammation-free EPM”; (**D**,**E**) group-2: “inflammation restricted to decidua”; (**F**,**G**) group-3: “inflammation restricted to the membranous trophoblast of chorion and the decidua”; (**H**,**I**) group-4: “inflammation in the connective tissue of chorion but not amnion”; and (**J**,**K**) group-5: “amnionitis”. Yellow circles indicate the measured area (≈40,000 μm^2^) in the innermost region of the EPM infiltrated by neutrophils from one section (**C**,**E**,**G**,**I**,**K**). Small blue circles indicate anti-BCL2A1-negative cells, whereas small red circles represent anti-BCL2A1-positive cells (**C**,**E**,**G**,**I**,**K**). All these images are based on the magnification setting × 100. These arrow point to the neutrophils that have infiltrated the innermost part of the EPM.

**Figure 2:**
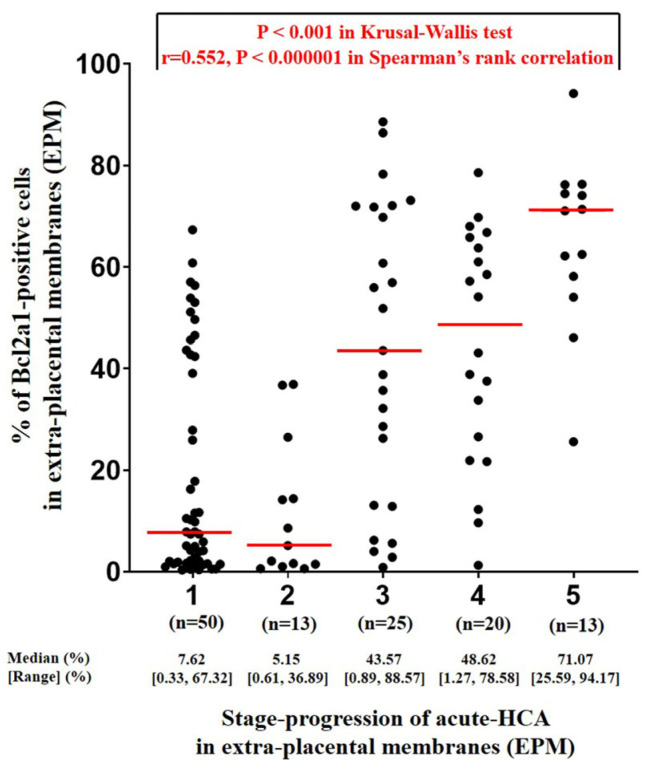
Digital image analysis using QuPath to determine the percentage of BCL2A1-positive cells according to the stage progression of acute HCA in EPM. Data are presented as median and range with individual values. Kruskal–Wallis test with Spearman’s rank correlation test was performed.

Figure 1 shows the representative H & E-stained images (B, D, F, H, and J) and QuPath IHC images of BCL2A1-positve cells (C, E, G, I, and K) according to the stage progression of acute HCA in the EPM. As acute HCA progresses in the EPM, both neutrophil infiltration and BCL2A1 expression can be observed migrating from the outermost layer, the decidua parietalis (Figure 1D,E), to the innermost layer, the amnion (Figure 1J,K). Moreover, digital image analysis using QuPath revealed that the percentage of BCL2A1-positive cells in the EPM significantly and progressively increases with the stage progression of acute HCA in the EPM, which is characterized by outside-in neutrophil migration (Spearman’s rank-correlation test, γ = 0.552, *p* < 0.000001; Kruskal–Wallis test, *p* < 0.001) (Figure 2). Interestingly, group-2 (i.e., inflammation restricted to decidua) shows no difference in the percent of BCL2A1-positive cells in the EPM compared to cases without inflammation (group-1) (median value; 5.15 vs. 7.62) (Figure 2). However, after the development of inflammation in the membranous trophoblast of chorion (group-3), there is a continuous increase in the percent of BCL2A1-positive cells according to the stage progression of acute HCA in the EPM (median value; group-3 vs. group-4 vs. group-5, 43.57 vs. 48.62 vs. 71.07) (Figure 2).

### 3.3. The Percentage of BCL2A1-Positive Cells in EPM, AFWBC, and Gestational Age at Delivery

**Figure 3:**
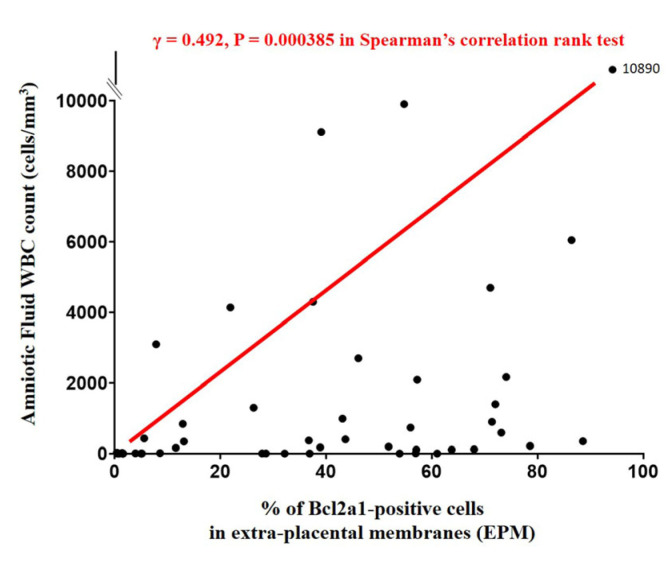
Relationship between the percentage of BCL2A1-positive cells in EPM and amniotic fluid WBC count. The percentage of BCL2A1-positive cells in EPM was positively and significantly correlated with amniotic fluid WBC count (cells/mm^3^). Spearman’s rank correlation test was performed.

**Figure 4:**
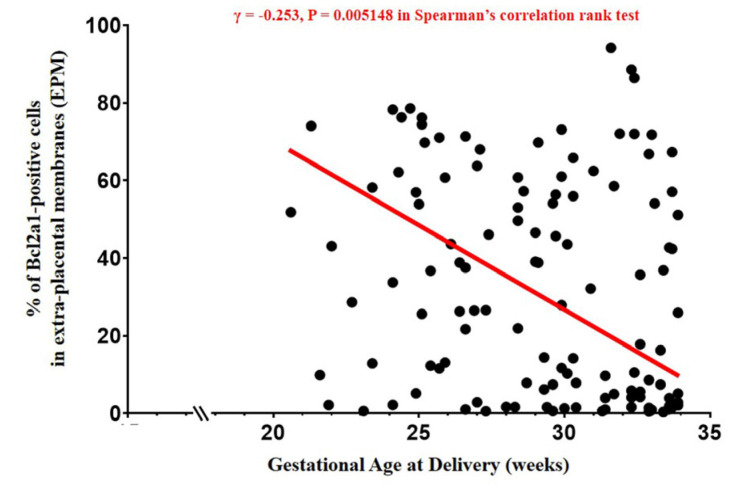
Relationship between the percentage of BCL2A1-positive cells in EPM and gestational age (GA) at delivery. The percentage of BCL2A1-positive cells in EPM was significantly decreased with increasing GA at delivery. Spearman’s rank correlation test was performed.

The percentage of BCL2A1-positive cells in the EPM and AFWBC count show a positive correlation (Spearman’s rank-correlation test, γ = 0.492, *p* = 0.000385) (Figure 3). Moreover, the percentage of BCL2A1-positive cells in the EPM continuously decreased with increasing GA at delivery (Spearman’s rank-correlation test, γ = −0.253, *p* = 0.005148) (Figure 4).

## 4. Discussion

### 4.1. Principal Findings

The intensity of BCL2A1 expression increases according to the stage progression of acute HCA, characterized by outside-in neutrophil migration in the EPM and the elevation of AFWBCs. Given the expression of BCL2A1 in neutrophils infiltrating EPM from a previous study [17], this finding suggests that BCL2A1 in the EPM may be a promising marker and target for acute HCA in spontaneous PTB.

### 4.2. Digital Image Analysis Using QuPath Can Calculate the Percentage of BCL2A1-Positive Cells as a Surrogate Marker for the Intensity of Neutrophils Infiltrating the Most Advanced Region of Acute HCA in EPM

The grade of acute HCA has been denigrated for the following reasons: (1) in various classification systems for acute HCA in the EPM, it has been simplistically divided into either mild or severe based solely on the number of infiltrating neutrophils [19,24], raising awareness that a simple binary distinction in grade cannot provide an accurate quantitative analysis; (2) the effect of grade on IAI intensity is less significant compared to that of stage in acute HCA [12,13], and this was likely due to the classification system being simplified into a binary format. It is not even known how the grade (i.e., the intensity of infiltrating neutrophils) varies according to the stage (i.e., the affected location by infiltrating neutrophils) of acute HCA in the EPM. Because neutrophils are not only a diagnostic criterion for acute HCA but also cells that need to be targeted for treatment in the EPM, it is essential to assess not only their location but also their intensity. Unfortunately, methods for objectively and quantitatively counting neutrophils in the EPM have rarely been reported. What is encouraging, however, is that we have previously reported, based on the study in both the human spontaneous PTB and NHP PTB model, that BCL2A1 is predominantly expressed in the cytoplasm of neutrophils in the context of the EPM of acute HCA [17]. Moreover, in the current study, QuPath was used to measure the percentage of BCL2A1-positive cells in EPM regions where acute HCA was most advanced. Identical-sized areas were used for measurement, making this approach highly objective. Therefore, we proposed that the intensity of BCL2A1 could serve as a surrogate marker for neutrophils infiltrating the EPM of acute HCA. However, to support this proposition, the quantitative results of BCL2A1 expression, as measured by QuPath, must align with findings from previous studies on the stage and grade of acute HCA based on neutrophil infiltration. Indeed, our current findings that the percentage of BCL2A1-positive cells increases according to the stage progression of acute HCA in the EPM (Figure 2) and positively correlates with the elevation of AFWBCs (Figure 3) are consistent with previous results, showing that as the stage of acute HCA in the EPM progresses, the severity of IAI increases [5]. Moreover, our current finding that the percentage of BCL2A1-positive cells in the EPM decreases progressively with increasing GA at delivery (Figure 4) is consistent with previous research demonstrating similar results as follows: (1) as the stage of acute HCA in the EPM progresses, GA at delivery decreases in the context of spontaneous PTB [25,26]; and (2) the inflammatory milieu of AF decreases in the same context of acute HCAs with GA at delivery [27].

### 4.3. BCL2A1 Could Enhance Its Value as a Therapeutic Target for Acute HCA

In a previous study, we identified BCL2A1 as the only gene consistently up-regulated among the top 10 genes from mRNA sequencing of the EPM during acute HCA in both human spontaneous PTB and the NHP PTB model [17]. Moreover, we demonstrated that treatment with the BCL2A1-specific inhibitor (i.e., ML214) in a chorio-decidua cell suspension cultured from rhesus macaques decreased the frequency of non-apoptotic neutrophils [17]. In contrast, treatment with the BCL2-specific, but not BCL2A1-specific, inhibitor (i.e., ABT-737) did not show any significant effect [17]. Notably, in spontaneous PTB, acute HCA is commonly observed, whereas inflammation in other maternal tissues or blood is extremely rare. Indeed, in the NHP PTB model, the concentration of uterotonic cytokines (i.e., IL-1beta and IL-8) in AF was higher in the IA LPS injection group (disease group) compared to the IA saline injection group (control group), whereas their concentrations in maternal plasma showed no difference (unpublished data). This is likely to be the case for BCL2A1 as well. Moreover, in normal tissues, BCL2A1 expression tends to be limited, though it is detected at low levels in other immune-related tissues (i.e., bone marrow and lymphoid tissues) and certain immune cells (i.e., neutrophils) [28]. Given that NSAIDs are neither safe nor durable therapeutic options during pregnancy due to fetal renal toxicity [29] and no other anti-inflammatory treatments for acute HCA are currently available, these findings further highlight the potential of BCL2A1 as a valuable therapeutic target for this condition.

### 4.4. Reconfirmation of Findings Consistent with the Results of Previous Studies

As shown in previous studies [5,11], the stage progression of acute HCA in the EPM was associated with earlier GA at delivery, lower birth weight, and a higher frequency of a 5 min Apgar score below 7. These findings suggest that a more detailed analysis of stage progression in acute HCA may better reveal a close association with prognostic factors such as GA at delivery.

### 4.5. Major Strengths and Limitations of This Study

Major strengths of this study are as follows: Firstly, the current study analyzed the stage progression of acute HCA in the whole sub-divisions of the EPM (i.e., decidua, the membranous trophoblast of chorion, the connective tissue of chorion, and amnion) in spontaneous PTB without maternal–fetal indication. Secondly, using QuPath, we sought to objectively assess the percentage of BCL2A1-positive cells—a marker previously shown to be valuable for neutrophil detection in the EPM of both human spontaneous PTB and the NHP PTB model [10]—through the random selection of equivalent areas from the most advanced inflammatory regions. Thirdly, this study focused on BCL2A1, which may be utilized as a therapeutic target in addition to an objective marker for diagnosing the intensity of acute HCA in the EPM. A limitation of this study is that we have not yet conducted research on BCL2A1 gene-related markers (i.e., cell-free fetal [cff] DNA and cff-RNA) in maternal plasma for predicting acute HCA characterized by BCL2A1 expression in the EPM. However, although we recently published a study suggesting that the neutrophil-to-lymphocyte ratio (NLR) in maternal blood may be a clue for identifying amnionitis, defined by neutrophils infiltrating the amnion—the innermost region of the EPM [30] —we have not yet found any studies that have consistently identified the same genetic markers for diagnosing acute HCA in the EPM from maternal plasma. Another limitation relates to the sample size. Although we obtained statistically significant results for both the primary and secondary outcomes with the current sample of 121 subjects in one institute, it may be necessary to validate these findings and other outcomes (i.e., the relationships between the percentage of BCL2A1-positive cells in the EPM and neonatal outcomes in the context of similar GAs at delivery) in a larger study population in the future.

### 4.6. Significance of This Study

This study is the first to apply QuPath in the analysis of acute HCA in the EPM, although few studies have applied QuPath in research related to placental vascular malperfusion in the extra-villous trophoblast and the vascular smooth muscle of villi in the placental disc [20,31]. Therefore, this application of QuPath to quantify the percentage of BCL2A1-positive cells in the EPM has paved the way for the objective assessment of the intensity related to neutrophils infiltrating the EPM regions where acute HCA was most advanced in addition to the staging of acute HCA in the EPM. This represents a groundbreaking advancement compared to the existing grade classification system [19,24], which previously simplified the quantitative analysis of neutrophil infiltration in H&E staining into only two groups. Such an objective assessment of the percentage of BCL2A1-positive cells using QuPath could help to accurately evaluate the impact of the inflammatory burden in acute HCA on GA at delivery and neonatal complications in spontaneous preterm birth. Additionally, it could aid in measuring the dosage and effectiveness of future targeted therapies. Based on the findings of a previous study [10] and our current research, we propose BCL2A1 as a surrogate marker for accurately assessing the intensity of acute HCA in the EPM and as a potential target for future targeted therapies.

### 4.7. Unanswered Questions and Further Study

It is not yet known whether the detection or elevation of BCL2A1-gene-related markers (i.e., cell-free fetal [cff] DNA and cff-RNA) in maternal plasma can be used to predict either the presence or the stage progression of acute HCA in the EPM. However, these challenges undoubtedly represent significant hurdles as they must be addressed through preclinical trials in NHP PTB models and clinical trials during human pregnancy. This kind of study will improve the value of non-invasive genetic inflammatory markers in maternal plasma for the identification of pregnant women at risk of spontaneous PTB. Moreover, it may also be applicable for selecting candidates for targeted therapy in clinical practice following successful preclinical research and clinical trials of BCL2A1-targeted therapy for acute HCA.

## 5. Conclusions

The intensity of BCL2A1 expression increases according to the stage progressionstage progression of acute histologic chorioamnionitis in the extra-placental membranes of spontaneous preterm birth. It may be necessary to validate the above results with a larger sample size in the future.

## Figures and Tables

**Table 1:** Clinical characteristics and pregnancy outcomes according to the stage progression of acute histologic chorioamnionitis (acute HCA) in extra-placental membranes (EPM).

	Group-1 ^†^	Group-2 ^†^	Group-3 ^†^	Group-4 ^†^	Group-5 ^†^	*p* Value ^††^
	41.3% (50/121)	10.7% (13/121)	20.7% (25/121)	16.5% (20/121)	10.7% (13/121)	
Maternal age, year (mean ± SD)	33.3 ± 4.6	34.0 ± 4.0	32.3 ± 4.1	33.1 ± 4.0	31.5 ± 4.8	NS (0.552)
Nulliparity	46.0% (23/50)	46.2% (6/13)	32.0% (8/25)	30.0% (6/20)	53.8% (7/13)	NS (0.498)
Preterm PROM as a cause of PTB	46.0% (23/50)	30.8% (4/13)	56.0% (14/25)	45.0% (9/20)	46.2% (6/13)	NS (0.695)
Male newborn	56.0% (28/50)	53.8% (7/13)	72.0% (18/25)	40.0% (8/20)	92.3% (12/13) ^a^	0.026
Cesarean delivery	46.0% (23/50)	23.1% (3/13)	24.0% (6/25)	30.0% (6/20)	46.2% (6/13)	NS (0.236)
GA at delivery, weeks (median [range])	31.6 [21.6–33.9]	29.1 [23.1–33.9]	29.9 [20.6–33.0]	27.2 [22.0–32.9] ^b^	25.1 [21.3–33.1] ^c^	<0.001
Birth weight, g (mean ± SD)	1615 ± 543	1375 ± 638	1409 ± 595	1160 ± 409 ^d^	1028 ± 549 ^e^	0.003
1 m Apgar score of <7	74.0% (37/50)	61.5% (8/13)	72.0% (18/25)	90.0% (18/20)	92.3% (12/13)	NS (0.202)
5 m Apgar score of <7	30.0% (15/50)	38.5% (5/13)	24.0% (6/25)	50.0% (10/20)	76.9% (10/13) ^f, g^	<0.05
Antenatal use of corticosteroids	74.0% (37/50)	76.9% (10/13)	80.0% (20/25)	85.0% (17/20)	76.9% (10/13)	NS (0.895)
Antenatal use of antibiotics	66.0 (33/50)	61.5% (8/13)	80.0% (20/25)	80% (16/20)	84.6% (11/13)	NS (0.394)
Antenatal use of tocolytics	52.0% (26/50)	76.9% (10/13)	84.0% (21/25)	90.0% (18/20) ^h^	92.3% (12/13)	<0.005

*GA*, gestational age; *NS*, not significant; *preterm PROM*, preterm premature rupture of membranes; *PTB*, preterm birth; *SD*, standard deviation. † Group-1: inflammation-free extra-placental membranes (EPM). † Group-2: inflammation restricted to decidua. † Group-3: inflammation restricted to the membranous trophoblast of chorion and the decidua. † Group-4: inflammation in the connective tissue of chorion but not amnion. † Group-5: amnionitis. ††: intergroup difference by Chi-square test (categorical variables) and Kruskal–Wallis test (continuous variables). a: *p* < 0.05 vs. group-4 (Fisher’s exact test with Bonferroni’s correction). b: *p* < 0.05 vs. group-1 (1-way ANOVA with post hoc Tukey test). c: *p* < 0.005 vs. group-1 (1-way ANOVA with post hoc Tukey test). d: *p* < 0.05 vs. group-1 (1-way ANOVA with post hoc Tukey test). e: *p* < 0.01 vs. group-1 (1-way ANOVA with post hoc Tukey test). f: *p* < 0.05 vs. group-1 (Fisher’s exact test with Bonferroni’s correction). g: *p* < 0.05 vs. group-3 (Fisher’s exact test with Bonferroni’s correction). h: *p* < 0.05 vs. group-1 (Fisher’s exact test with Bonferroni’s correction).

## Data Availability

The raw data presented in this study can be obtained upon reasonable request to Chan-Wook Park at hwpark0803@gmail.com.
